# Gamma probe and ultrasound guided fine needle aspiration cytology of the sentinel node (GULF) trial - overview of the literature, pilot and study protocol

**DOI:** 10.1186/s12885-017-3236-2

**Published:** 2017-04-12

**Authors:** Charlotte M.C. Oude Ophuis, Lisa (Linetta) B. Koppert, Cécile de Monyé, Carolien H.M. van Deurzen, Senada Koljenović, Alexander C.J. van Akkooi, Cornelis (Kees) Verhoef, Dirk J. Grünhagen

**Affiliations:** 1 Department of Surgical Oncology, Erasmus MC Cancer Institute, Groene Hilledijk 301, 3075 Rotterdam, EA The Netherlands; 2 Department of Radiology, Erasmus MC Cancer Institute, Groene Hilledijk 301, 3075 Rotterdam, EA The Netherlands; 3 Department of Pathology, Erasmus Medical Center, Wytemaweg 80, 3015 Rotterdam, CN The Netherlands; 4 Department of Surgical Oncology, Netherlands Cancer Institute – Antoni van Leeuwenhoek, Plesmanlaan 121, 1066 Amsterdam, CX The Netherlands

**Keywords:** Sentinel lymph node biopsy, Ultrasonography, Fine-needle biopsy, Melanoma, Breast Neoplasms, Minimally invasive surgical procedures

## Abstract

**Background:**

Sentinel node (SN) biopsy (SNB) detects clinically occult metastases of breast cancer and melanoma in 20–30%. Wound infections, seroma and lymph edema occur in up to 10%. Targeted ultrasound (US) of the SN, (with fine needle aspiration cytology (FNAC) if appropriate) has been investigated as a minimally invasive alternative, but reported sensitivity rates are too low to replace SNB. Our hypothesis is that the use of a handheld gamma probe concomitant with US may improve sensitivity.

Our aim is to provide an overview of the current literature on preoperative nodal staging of clinical N0 melanoma patients, report on a pilot, and present a study protocol for a minimally invasive alternative to the SNB: **G**amma probe and **Ul**trasound guided **F**ine needle aspiration cytology of the sentinel node (GULF trial).

**Methods:**

The GULF trial is a multicenter open single arm observational trial. Newly diagnosed cT1b-4N0M0 cutaneous melanoma or cT1-3N0M0 breast cancer patients, aged >18 years, presenting for SNB are eligible. 120 patients will be included for preoperative targeted gamma probe guided US and FNAC of the SN. Afterwards all patients proceed to surgical SNB. Primary endpoint is the sensitivity of FNAC. Secondary endpoints include SN identification rate and the histopathological compatibility of Core Needle Biopsy and FNAC vs. SNB. Secondary endpoints were investigated in a pilot with 10 FNACs and marker placements, and 10 FNACs combined with Core Needle Biopsy.

**Results:**

A pilot in 20 patients showed that SN identification rate was 90%, supporting the feasibility of this technique.

**Discussion:**

There is broad experience with US (in combination with FNAC) prior to SNB, but sensitivity and specificity are too low to completely abandon SNB. Promising alternative techniques potentially will replace SNB in the future but more evidence is needed in the form of prospective studies. Accurate identification of the SN for US-FNAC has been proven feasible in our pilot. When adequate sensitivity can be reached, US-FNAC provides a minimally invasive alternative for the surgical SNB procedure.

**Trial registration:**

The GULF trial is registered in the Netherlands Trial Registry (NTR), ID: NRT5193. May 1st 2015.

**Electronic supplementary material:**

The online version of this article (doi:10.1186/s12885-017-3236-2) contains supplementary material, which is available to authorized users.

## Background

### Sentinel node biopsy

With the introduction of sentinel node (SN) biopsy (SNB) as a less invasive alternative to elective lymph node dissection for melanoma and breast cancer with clinically negative lymph nodes, this has become the gold standard for adequate staging. Although less invasive than an elective lymph node dissection, SNB is still associated with some potential morbidity. Morbidity occurs in up to 10% of patients; wound infections and seroma are the most frequently seen complications [1, 2]. Rarely lymph edema is seen after SNB. Around 70–80% of SNB’s are tumor negative after histological assessment, these patients cannot benefit from the SNB procedure. In that light the morbidity of a surgical SNB procedure is deemed considerable, and any less invasive procedure, if accurate enough, would be preferred.

The detection rate of submicrometastases has increased considerably in the past decades; adaptation of the melanoma and breast cancer SN sectioning protocols and use of standard immunohistochemistry staining enabled pathologists to detect even the smallest tumor deposits accurately [3–8]. This has clear clinical implications; more patients are diagnosed as SN positive and will be offered a completion lymphadenectomy (CLND) [6, 8]. It is questionable whether this morbid surgical procedure is justified in cases with minimal SN tumor burden [9], as several retrospective melanoma studies and recently the prospective DeCOG study have shown that survival for this group of melanoma patients is similar to SN negative patients [10–13]. In breast cancer, presence of isolated tumor cells (≤0.2 mm) or micrometastases (>0.2 ≤ 2.0 mm) is associated with a slightly worse prognosis [7, 14], but its clinical relevance is debated as well [15–17], and CLND is omitted in certain groups of patients with a positive SN [18].

Prospective studies currently investigating the therapeutic value of CLND in melanoma are the EORTC-1208MG (Minitub) [9], including patients with minimal SN tumor burden only, and the MSLT2, which included all SN positive patients [19]. Parallel to this, certain adjuvant therapy trials (EORTC 18071, EORTC 1325, Combi-AD) recruit stage IIIA patients only in case of ≥1 mm SN tumor burden [20–22].

Primary results from the EORTC 18071 show that SN positive patients benefit the most from adjuvant treatment measured as recurrence free survival at 3 years [21, 22]. In this light, it remains worthwhile to keep selecting patients for adjuvant therapy in trial setting and/or CLND based on nodal staging, and a cut-off for detection of (sub) micrometastases (<1.0 mm) may aid in prevention of overtreatment in low risk patients.

### Ultrasound guided fine needle aspiration cytology

Ultrasound (US) guided fine needle aspiration cytology (FNAC) or core needle biopsy (CNB) may provide a good minimally invasive alternative to SNB. In breast cancer patients screening US of the regional lymph node basin is part of the preoperative staging process; this way up to 17% of patients undergo axillary lymph node dissection immediately and are spared a SNB [23, 24]. Melanoma patients do not routinely undergo a preoperative US of the regional lymph node basin, due to previously reported poor identification rates of occult lymph node metastases with US and FNAC [25, 26]. Several studies have been conducted in order to analyze if US (with FNAC or CNB) can replace SNB, but thus far reported sensitivity rates for US vary considerably, ranging between 9 and 94% [27, 28].

For the current study we will focus on melanoma, as the therapeutic value of both SNB and CLND are debated, and alternatives for SNB are more limited for this type of cancer.

In our search for a reliable and accurate minimally invasive alternative to SNB for staging of clinical N0 melanoma patients, we examined the current available literature and performed a systematic search of all major databases to explore whether other methods than US guided FNAC may have proven adequate alternatives to SNB.

### Literature overview

All relevant studies on US imaging of regional lymph nodes in melanoma patients scheduled for SNB are displayed in Table 1 (search details are given in Additional file 1). Some of the studies mentioned in Table 1 are overlapping; the studies from Voit et al. [29–33] concern the same database with more inclusions over time. In the studies that performed US prior to lymphoscintigraphy, sensitivity rates were low, ranging from 4.7% to 39%, and specificity rates were high, ranging from 86% to 100%. Two studies did not mention the exact timing of US in relation to lymphoscintigraphy; Hocevar et al. reached a sensitivity and specificity of 71% and 84%, and Testori et al. reached a sensitivity of 94% and 90%. In the studies that performed a targeted US (i.e. US of the marked “SN” area on the skin after lymphoscintigraphy), sensitivity ranged from 22% to 100%, and specificity ranged from 62% to 100% (Table 1).

**Table 1:** Studies of Ultrasound Imaging of Regional Lymph Nodes in Melanoma Patients Scheduled for Sentinel Node Biopsy

Author, year	Study Design	*N*	US Setting	FNAC/other technique	Sens (%)	Spec (%)	PPV (%)	NPV (%)
Rossi [61], 2000	Not mentioned	69	Pre-lympho	No	33	100	100	86
Rossi [25], 2003	Prospective, monocentric	125	Pre-lympho	FNAC if US suspicious	US alone: Not mentioned	-	-	-
					US-FNAC: 39	100	100	85
Hocevar [62], 2004	Prospective, monocentric	57	unknown	FNAC if US suspicious	US alone: 71	84	59	90
					US-FNAC: Not mentioned	-	-	-
Testori [63], 2005	Retrospective, monocentric	88	unknown	No	94	90	64	99
Starritt [64], 2005	Prospective, monocentric	31 all SN +	Post-lympho	No	NA	NA	NA	NA
Voit [65], 2006	Prospective, monocentric	127	Post-lympho	FNAC if US suspicious	US alone: 79	72	53	90
					FNAC alone: 59	100	100	85
					US-FNAC: 82	72	54	91
Van Rijk [26], 2006	Retrospective, monocentric	107	Pre-lympho	FNAC if US suspicious	US alone: 34	87	-	-
					US-FNAC: 4.7	100	-	-
Sibon [66], 2007	Prospective, monocentric	131	Pre-lympho	No	9	96	43	-
Kunte [67], 2009	Prospective, monocentric	25	Pre- and post-lympho	No	33	100	100	88
Voit [30], 2009	Prospective, monocentric	400	Post-lympho	FNAC if US suspicious	65	99	93	92
Sanki [60], 2009	Prospective, monocentric	716	Post-lympho	No	33	97	60	88
De Giorgi [36], 2010	Prospective monocentric	15	Post-lympho	Standard CEUS	CEUS: 100	62	55	100
Voit [31], 2010	Prospective monocentric	400	Post lympho	FNAC if US suspicious	All Berlin criteria combined: 82	80	52	94
Hinz [58], 2011	Prospective monocentric	81	Pre and post lympho	No	22	100	100	96
Chai [68], 2012	Retrospective monocentric	325	Pre-lympho	FNAC if US suspicious	34	86	37	84
Marone [69], 2012	Prospective monocentric	623	Pre-lympho	No	15	100	100	87
Pilko [70], 2012	Retrospective Monocentric	405	Pre-lympho	FNAC if US suspicious	Not mentioned	-	-	-
Stoffels [71], 2012	Retrospective Monocentric	221	Pre-lympho	FNAC if US suspicious	14	97	100	97
Hinz [72], 2013	Retrospective Monocentric	20	Pre-lympho &, pre PET-CT	No If US malig. Direct LND	12	100	100	74
Ulrich [73], 2014 *In German*	Prospective monocentric	800	Post lympho	FNAC if US suspicious	US-FNAC: 56	99	92	89
Voit [32], 2014	Prospective monocentric	1000	Post lympho	FNAC if US suspicious	US alone: 71	-	-	-
					US-FNAC: 51	99	99	89
Voit [33], 2016	Prospective monocentric	1000	Post lympho	FNAC if US suspicious	US alone: 71	-	-	-
					US-FNAC: 51	99	99	89

Overview of studies reporting on ultrasound imaging of regional lymph nodes in melanoma patients prior to sentinel node biopsy. Abbreviations: *US* ultrasound, *FNAC* fine needle aspiration cytology, *CEUS *contrast enhanced ultrasound﻿, *Sens* sensitivity, *Spec* specificity, *PPV* positive predictive value, *NPV* negative predictive value, *lympho* lymphoscintigraphy, *NA* not applicable

Besides US and targeted US with FNAC prior to SNB, several groups have focused on development of new imaging techniques for examination of the SN/lymph nodes and detection of SN tumor deposits, such as sonoelastography [34, 35], contrast enhanced US [36], and multispectral optoacoustic tomography (MSOT) [37] (Table 2). Sonoelastography measures tissue consistency; which can be visualized on top of US images using different color shades; red indicating soft tissue, and blue indicating rigid tissue [34, 35]. As metastases tend to be more solid than normal lymph node tissue regions of interest for FNAC can be identified. For contrast enhanced US an intravenous contrast agent is applied to detect possible areas of hyperperfusion or hypoperfusion; indicating potential metastatic lesions [36]. These techniques reached a high sensitivity for identification of SN metastases (Table 2).

**Table 2:** Pilot studies on novel techniques for pre-operative non-invasive detection of melanoma metastases in lymph nodes

Author, yr	Topic	No. of patients	Technique	Sens (%)	Spec (%)
Hinz [72], 2013	Elastography	36	US + power Doppler:	81	76
			Elastography:	91	76
			Combined:	95	76
Ogata [35], 2014	Elastography	12	US:	77	57
			Elastography:	100	71
Stoffels [37], 2015	MSOT and indocyanin green	20	MSOT	100	48.6

Overview of pilot studies investigating non-invasive detection of melanoma lymph node metastases

Abbreviations: *yr.* year, *Sens* sensitivity, *Spec* specificity, *US* ultrasound, *MSOT* multispectral optoacoustic tomography

Two recently developed techniques for improved SN identification peri-operatively are SPECT-US [38, 39], and near infrared light fluorescence imaging [40–44]. SPECT-US displays the location of a radio-active SN in the US images; making it easier for the surgeon to locate SN’s in anatomically challenging area’s such as the cervical and occipital area; or to guide radiologists for FNAC [38, 39]. Near infrared light fluorescence imaging is conducted with Indocyanine green as tracer, which can be combined with 99Tc nano-colloids to form a hybrid tracer [41]. Intraoperative identification is similarly accurate to 99Tc-colloid; and particularly helpful for SN localization in the cervical and occipital area, where overprojection from the 99TC-colloid injection site is a common obstacle. Preoperative (transcutaneous) SN identification has reached lower identification rates, due to the limited penetration depth of the fluorescent tracer [45].

Summarizing, few US imaging studies have used a method to accurately identify the SN prior to US examination and FNAC. This may have contributed to lower than expected sensitivity rates for detection of SN metastases in studies where this was not applied. It explains why to date no alternative method for SN staging has been adopted in daily clinical practice, and the need for such a method remains.

### Rationale for a new trial

#### SN identification

To overcome the problem of suboptimal identification of the SN, we hypothesize that use of a handheld gamma probe (Geiger teller) to detect the SN post lymphoscintigraphy may further aid the radiologist in accurately identifying the SN for ultrasound guided FNAC. Several pilot studies have been performed using this technique in breast cancer patients; correct localization of the SN occurred in 75% - 100% [46–49]. This formed the rationale for the GULF Trial (**G**amma probe and **Ul**trasound guided **F**ine needle aspiration cytology of the sentinel node).

#### Cytology or histology?

In order to reach the sample size needed for proof of concept with accurate power and within an acceptable term, both melanoma patients and breast cancer patients will be included in the GULF trial. The SN procedure is uniformly applied for both melanoma and breast cancer, and breast cancer patients may equally benefit from a minimally invasive alternative for the SN. All patients will undergo FNAC. Since metastatic size may have clinical implications for breast cancer patients [18], a subset of 10 breast cancer patients will undergo CNB additionally after FNAC. This allows for a comparison of results between CNB, FNAC and SNB.

#### Hypotheses GULF trial

We hypothesize that a sensitivity of 90% with a 95% confidence interval of 80% - 100% is achievable. Secondly, we expect that a SN identification rate of more than 75% is feasible.

#### Study aims

- To present a study protocol for a minimally invasive alternative to the sentinel node biopsy (GULF trial), with as primary objective to determine whether an acceptable sensitivity for US and gamma probe guided FNAC can be achieved.
- Secondary objective is 1) the identification rate of the SN and 2) the histological results of CNB versus FNAC and versus SNB.

Prior to starting the GULF-trial, we had to prove the concept of adequate identification of the SN. A pilot study focusing on the adequate detection rate of the SN was conducted.

### Pilot

After approval of the Ethical Review Board a pilot was performed in 20 patients presenting at the Erasmus MC Cancer Institute. All patients underwent gamma probe guided US-FNAC after written informed consent. In the 10 first melanoma patients additional metallic marker placement (O-Twist-Marker, BIP) was performed after local infiltration of the skin and surrounding tissue with 1-10 mL lidocaine 2%. Correct identification of the SN was assessed by examining the excised SN (s) on presence of the marker. Separately, in the first 10 breast cancer patients CNB was performed after FNAC with a 14G needle, after local infiltration similar to marker placement. CNB was done for assessment of concordance with FNAC results and to detect potential superiority of either technique.

All patients proceeded to OR for SNB, which was performed according to the triple technique: preoperative 99Tc lymphoscintigraphy <24 h prior to surgery, intradermal injection of patent blue near the primary tumor site prior to first incision, and peroperative use of a handheld gamma-probe to locate SN (s) [50, 51], Lymph nodes were considered SN when radioactive and/or blue. A marker was retrieved from the SN in 9 out of 10 patients; which meant the SN identification rate was 90%. CNB samples were investigated on presence of lymphoid tissue. This was present in 6 out of 10 patients. 40% of CNBs was not representative. In comparison: FNAC color staining was representative in 19 out of 20 patients (95%), and FNAC immunohistochemistry staining was representative in 14 out of 20 patients (70%).

During the pilot study no safety issues occurred. In the second enrolled study patient none of the 2 placed markers were found at histopathological examination of the SN and in another patient only 1 of 2 placed markers was found. A detailed shoulder X-ray confirmed the markers were still in situ in both patients. In the latter patient the X-ray images were suggestive of marker displacement towards mamma tissue; this was probably due to intraoperative displacement of the marker during SN removal.

Considering the positive results from this pilot, the study will be continued with an expansion of the pilot population in order to reach a sufficient sample size according to the presented study protocol.

## Methods

### GULF design

**Fig. 1:**
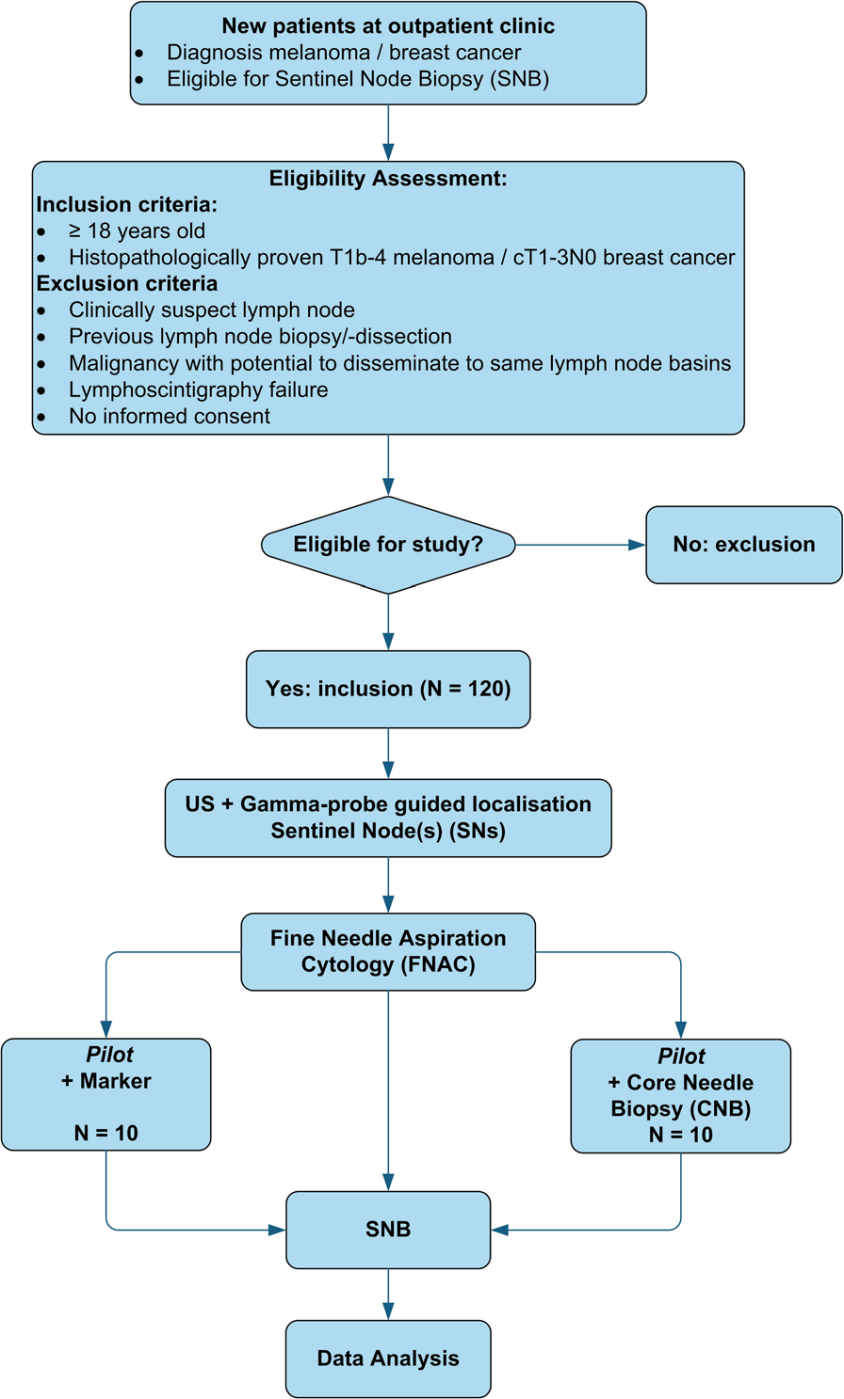
Study Flowchart GULF Trial

Patients with a newly diagnosed cT1b-4N0M0 cutaneous melanoma or cT1-3N0M0 breast cancer presenting at the outpatient clinic of the Erasmus MC Cancer Institute, and the Netherlands Cancer Institute – Antoni van Leeuwenhoek (only melanomas) will be assessed for inclusion. All patients will undergo US and gamma probe guided FNAC of the SN. The pilot patients received additional marker placement (*n* = 10) for identification purposes, or additional CNB (*n* = 10) for assessment of potential benefit of CNB (i.e. histology and size measurement possible) (Fig. 1).

### Study population

#### Inclusion criteria

Age ≥ 18 years, new diagnosis of cT1b-4N0M0 cutaneous melanoma or cT1-3N0M0 breast cancer.

Prior to start of any study related procedure, written informed consent must be given according to ICH/GCP and national legislation.

#### Exclusion criteria

Clinically suspect lymph node, other known malignancy with potential to disseminate to axillary or groin lymph node basins, prior lymph node biopsy, no SN visible at lymphoscintigraphy/not identifiable with gamma probe.

### Study procedures

#### US-FNAC

All patients will be admitted to the surgical ward on the day of surgery. First, a lymphoscintigram <24 h prior to SNB will be performed, as is standard procedure. Following successful lymphoscintigraphy (i.e. SN (s) is/are visible) the study procedures can start. A dedicated radiologist will perform US imaging of the lymph node basin where a SN or multiple SNs was/were identified by lymphoscintigraphy. The exact location of the SN (s) will be determined using a handheld gamma probe, and by combination with US; the assumed SN (s) will be visualized (being a visible lymph node at the center of the hotspot found with the gamma probe). FNAC will be performed of all visualized assumed SN (s). In case of multiple SNs in one lymph node basin or multiple draining lymph node basins with an SN in every basin (for instance a melanoma on the back draining to both axilla and groin), FNAC will be performed of all lymph nodes pointed out as primary tier SN by the nuclear medicine specialist. For FNAC 1–4 cortical samples will be taken. Whenever additional clearly suspect lymph nodes are visible, the radiologist will perform FNAC from these nodes as well, as is standard of care. All samples will be transported to the pathology lab for analysis. US findings will be recorded according to the Berlin morphologic Criteria to create uniformity in recording per center [30]. After FNAC, all patients will proceed to the operating room for SNB according to standard procedure (as described in the Pilot section). Lymph nodes were considered SN when radioactive and/or blue. No diagnostic procedure or treatment is postponed or elongated. No additional visits to the outpatient clinic are required.

#### Endpoints

*Primary endpoint:* Primary outcome is the sensitivity of gamma probe and US guided CNB or FNAC.

*Secondary endpoints:* Secondary outcome is 1) the identification rate of the SN 2) the histological results of CNB versus FNAC and versus SNB.

Ad 1) an identification rate of at least 75% is deemed acceptable (concordant with literature). This has been proven feasible in the pilot study.

### Statistical considerations

#### Sample size and accrual

Based on retrospective data, the prevalence of metastatic SNs is expected to be 30%. Our gold standard is the histological outcome of SNB (absence or presence and size of metastases in the SN). Submicrometastases (i.e. <0.1 mm at any site or 0.4 mm subcapsular) in melanoma patients, and isolated tumor cells (i.e. ≤0.2 mm) in breast cancer patients will be considered negative: a negative FNAC is accepted in these cases. Based on previous reports, we expect to find around 10% of these submicrometastases and isolated tumor cells in both melanoma and breast cancer patients [52, 53]. Considering this, the maximum achievable sensitivity of FNAC will be 90%. For this sensitivity, and a 95% confidence interval of 80–100% (With a two-sided significance level α = 0.05 and power 1 – β = 0.8), the required sample size is 116 considering a 30% prevalence of metastatic SNs. Around 3% of patients are expected to have a negative lymphoscintigram: the sample size will be increased to 120 patients.

With an average accrual rate of 60 patients per year, maximum accrual will be met at 2 years post start of study.

### Statistical analysis plan

The main analysis addressing the primary endpoint will be performed after inclusion of all 120 patients. No interim analysis is planned for this endpoint.

### Ethical considerations

This study has been approved by the Erasmus MC medical-ethical committee. The study will be conducted according to the principles of the Declaration of Helsinki and in accordance with national and regional legislation, guidelines, regulations and acts.

## Discussion

Currently SNB is the most important staging procedure for clinically N0 melanoma patients, especially in the light of trial participation for adjuvant therapies based on N-status [20, 21, 54]. The therapeutic role of SNB for melanoma is still under debate [55–57]. Considering the fact that this is a surgical staging procedure associated with complications in up to 10% of patients, our group sought to investigate a more minimally invasive alternative.

The ongoing improvement of imaging techniques (i.e. more accurate and detailed US imaging) and increased experience with FNAC renders combined US-FNAC as a high potential minimally invasive alternative for surgical SNB [31, 32]. Correct transcutaneous identification of the SN forms the main obstacle for broad application of this technique as this is key in obtaining reliable FNAC.

The current study aims to give an overview of the current melanoma literature, report a pilot and present a study protocol for a minimally invasive technique to investigate the SN using gamma probe guided US-FNAC.

### Overview of the literature

The studies presented provide evidence that it is difficult to detect clinically occult lymph node metastases in melanoma patients, and although some studies have achieved high sensitivity and specificity rates, these results have not been reproduced by other groups. There are many differences between the reported studies; namely retrospective vs. prospective study setting; US prior to lymphoscintigraphy vs. targeted US after lymphoscintigraphy; the number of persons performing US and their expertise; variation in US morphology criteria used to discriminate between benign and suspicious or malignant lymph nodes; and use of FNAC or not. Al these factors will have contributed to the outcome of these studies. It is interesting to see that sensitivity rates are low in the studies that performed an US of the entire lymph node basin without knowing the location of the SN (s), but that even in the studies were targeted US of the SN area was applied, sensitivity rates could be as low as 22% [58] and as high as 82% [31] or even a perfect 100% [36] as well. Thompson et al. proposed a possible explanation for these disparate results; many of the micrometastases present in SNs are too small to be detected by the US-equipment used [59, 60]. However, Voit et al. demonstrated that it was possible to successfully perform a FNAC in a lesion as small as 0.4 mm. Nevertheless, most smaller SN metastases will be overlooked by US and/or missed by FNAC. The question is if this has any clinical implications.

As long as US-imaging is limited by a detection limit, and alternative imaging techniques are tested in pilot settings, the need for a reliable, minimal invasive easy to perform and replicate method to assess SN status remains. Hence the presentation of the GULF trial study protocol here.

### Pilot

Our pilot results show that correct identification of the SN for FNAC was possible in 90%, and that the sampled material was representative in 95% of FNAC samples. CNB was representative in only 60%. This confirms that the described technique for targeted US-FNAC of the SN is feasible. CNB will not be added to the study procedure considering the low rate of representative tissue in the pilot phase.

If an acceptable sensitivity can be achieved for FNAC, patients can proceed to undergo radical lymph node dissection immediately in case of positive FNAC, bypassing the SNB procedure. When the FNAC sample is negative, surgeons can choose to perform a SNB or continue with only surgical excision of the primary tumor and monitoring of potential lymph node involvement at follow up visits. This way up to 80% of patients eligible for SNB can be spared this invasive procedure and the risk of morbidity related to this procedure. Furthermore, for melanoma patients this would mean that general anesthesia is no longer needed, as WLE can be performed under local anesthesia. Ultimately operative nodal staging may become completely obsolete.

## Conclusions

The literature on pre-operative assessment of regional lymph nodes with US in clinically N0 melanoma patients is disparate. Targeted US of the SN area in combination with FNAC or other new techniques has potential to become a minimally invasive alternative for the SNB, however, findings need to be replicated in prospective clinical trials first. A pilot with gamma probe guided US-FNAC show that accurate SN identification in up to 90% of patients is feasible. Our group presents a study protocol of the *Gamma probe and ULtrasound guided Fine needle aspiration cytology of the sentinel node Trial* (GULF trial) as a potential improvement to the reported US-FNAC techniques and ultimately even a possible replacement of the SNB.

## Additional file

Additional file 1: Data gives an overview of the number of found articles per literature database, and specific entry terms are provided. (PDF 14 kb)

## Abbreviations

CLND: Completion lymphadenectomy
CNB: Core needle biopsy
FNAC: Fine needle aspiration cytology
GULF: Gamma probe and ultrasound guided fine needle aspiration cytology of the sentinel node trial”
ICH-GCP: International Conference on Harmonisation - Good Clinical Practice
MSOT: Multispectral optoacoustic tomography
SN: Sentinel node
SNB: Sentinel node biopsy
SPECT: Single photon emission computed tomography
US: Ultrasound
