# Erratum to: gamma probe and ultrasound guided fine needle aspiration cytology of the sentinel node (GULF) trial - overview of the literature, pilot and study protocol

**DOI:** 10.1186/s12885-017-3363-9

**Published:** 2017-05-30

**Authors:** Charlotte M.C. Oude Ophuis, Lisa B. Koppert, Cécile de Monyé, Carolien H.M. van Deurzen, Senada Koljenović, Alexander C.J. van Akkooi, Cornelis Verhoef, Dirk J. Grünhagen

**Affiliations:** 1 Department of Surgical Oncology, Erasmus MC Cancer Institute, Groene Hilledijk 301, 3075 Rotterdam, EA The Netherlands; 2 Department of Radiology, Erasmus MC Cancer Institute, Groene Hilledijk 301, 3075 Rotterdam, EA The Netherlands; 3 Department of Pathology, Erasmus Medical Center, Wytemaweg 80, 3015 Rotterdam, CN The Netherlands; 4 Department of Surgical Oncology, Netherlands Cancer Institute – Antoni van Leeuwenhoek, Plesmanlaan 121, 1066 Amsterdam, CX The Netherlands

## Erratum

After the publication of this work [1] it was noticed that the Authors’ initials for ‘Lisa (Linetta) B. Koppert’ and ‘Cornelis (Kees) Verhoef’ were incorrect on PubMed. On PubMed, the author ‘Lisa (Linetta) B. Koppert’ appeared as ‘Koppert LL’, but should appear as ‘Koppert LB’. The author ‘Cornelis (Kees) Verhoef’ appeared as ‘Verhoef CK’, but should appear as ‘Verhoef C’. The errors were due to the additional names in parentheses being tagged in the XML as a ‘givenname’. These names in the parentheses have been removed to correct the initials on PubMed. The original article has been corrected.
