# Supplementary material for: Gamma probe and ultrasound guided fine needle aspiration cytology of the sentinel node (GULF) trial - overview of the literature, pilot and study protocol
Source: BMC Cancer. 2017 Apr 12;17:258. doi: 10.1186/s12885-017-3236-2 (PMC5389093; doi:10.1186/s12885-017-3236-2)
Supplement: Additional file 1: — Data gives an overview of the number of found articles per literature database, and specific entry terms are provided. (PDF 14 kb) [file 12885_2017_3236_MOESM1_ESM.pdf]

Literature Search 22 april 2016

Subject: Melanoma echography lymph nodes

Database	Number of studies	Number of unique studies
Embase.com	347	340
Medline Ovid	213	28
Web of science	286	122
Cochrane	5	0
PubMed publisher	5	4
Google scholar	100	69
**Total**	**956**	**563**

Embase.com n=347

('melanoma'/exp OR (melanom*):ab,ti) AND ('lymph node'/exp OR 'lymph node biopsy'/exp OR 'lymph node metastasis'/exp OR (lymph-node* OR sentinel-node* OR SLNB OR SLN OR SLNs OR SN):ab,ti) AND ('echography'/exp OR ultrasound/de OR (echogra* OR ultraso* OR (us NEXT/1 (guid* OR examin* OR imag*)) OR preoperative-US OR sonogra* OR optoacoust*):ab,ti) AND ('diagnostic accuracy'/exp OR 'diagnostic test accuracy study'/de OR 'diagnostic error'/exp OR 'predictive value'/de OR 'diagnostic value'/de OR 'sensitivity and specificity'/exp OR 'early diagnosis'/de OR 'reproducibility'/de OR 'observer variation'/de OR (((diagnos* OR ultrasound OR us) NEAR/6 (accurac* OR error* OR metast* OR abilit* OR value OR improv* OR mis OR missed)) OR ((Micrometasta* OR metasta* OR sentin* OR sn OR sln) NEAR/6 (identif* OR detect*)) OR (false NEXT/1 (positive* OR negative*)) OR predictive-value* OR npv OR ppv OR sensitiv* OR specific* OR (early NEAR/3 (diagnos* OR detect*)) OR reproducib* OR Misdiagnos* OR ((observer* OR interobserver* OR intraobserver*) NEAR/3 (varia* OR bias))):ab,ti) NOT ([Conference Abstract]/lim OR [Letter]/lim OR [Note]/lim OR [Editorial]/lim)

Medline Ovid n=213

("melanoma"/ OR (melanom*).ab,ti.) AND ("Lymph Nodes"/ OR "Sentinel Lymph Node Biopsy"/ OR (lymph-node* OR sentinel-node* OR SLNB OR SLN OR SLNs OR SN).ab,ti.) AND (exp "Ultrasonography"/ OR "Ultrasonography".xs. OR Ultrasonics/ OR (echogra* OR ultraso* OR (us ADJ (guid* OR examin* OR imag*)) OR preoperative-US OR sonogra* OR optoacoust*).ab,ti.) AND (exp "Diagnostic Errors"/ OR "diagnostic value"/ OR exp "sensitivity and specificity"/ OR exp "early diagnosis"/ OR "Reproducibility of Results"/ OR (((diagnos* OR ultrasound OR us) ADJ6 (accurac* OR error* OR metast* OR abilit* OR value OR improv*)) OR ((Micrometasta* OR metasta* OR sentin* OR sn OR sln) ADJ6 (identif* OR detect*)) OR (false ADJ (positive* OR negative*)) OR predictive-value* OR npv OR ppv OR sensitiv* OR specific* OR (early ADJ3 (diagnos* OR detect*)) OR reproducib* OR Misdiagnos* OR ((observer* OR interobserver* OR intraobserver*) ADJ3 (varia* OR bias))).ab,ti.) NOT (letter OR news OR comment OR editorial OR congresses OR abstracts).pt.

Cochrane n=5

((melanom*):ab,ti) AND ((lymph-node* OR sentinel-node* OR SLNB OR SLN OR SLNs OR SN):ab,ti) AND ((echogra* OR ultraso* OR (us NEXT/1 (guid* OR examin* OR imag*)) OR preoperative-US OR sonogra* OR optoacoust*):ab,ti) AND ((((diagnos* OR ultrasound OR us) NEAR/6 (accurac* OR error* OR metast* OR abilit* OR value OR improv* OR mis OR missed)) OR ((Micrometasta* OR metasta* OR

sentin* OR sn OR sln) NEAR/6 (identif* OR detect*)) OR (false NEXT/1 (positive* OR negative*)) OR predictive-value* OR npv OR ppv OR sensitiv* OR specific* OR (early NEAR/3 (diagnos* OR detect*)) OR reproducib* OR Misdiagnos* OR ((observer* OR interobserver* OR intraobserver*) NEAR/3 (varia* OR bias))):ab,ti)

Web of science n=286

TS=(((melanom*)) AND ((lymph-node* OR sentinel-node* OR SLNB OR SLN OR SLNs OR SN)) AND ((echogra* OR ultraso* OR (us NEAR/1 (guid* OR examin* OR imag*)) OR preoperative-US OR sonogra* OR optoacoust*)) AND ((((diagnos* OR ultrasound OR us) NEAR/5 (accurac* OR error* OR metast* OR abilit* OR value OR improv* OR mis OR missed)) OR ((Micrometasta* OR metasta* OR sentin* OR sn OR sln) NEAR/5 (identif* OR detect*)) OR (false NEAR/1 (positive* OR negative*)) OR predictive-value* OR npv OR ppv OR sensitiv* OR specific* OR (early NEAR/2 (diagnos* OR detect*)) OR reproducib* OR Misdiagnos* OR ((observer* OR interobserver* OR intraobserver*) NEAR/2 (varia* OR bias)))))

PubMed publisher n=5

("melanoma"[mh] OR (melanom*[tiab])) AND ("Lymph Nodes"[mh] OR "Sentinel Lymph Node Biopsy"[mh] OR (lymph-node*[tiab] OR sentinel-node*[tiab] OR SLNB OR SLN OR SLNs OR SN)) AND ("Ultrasonography"[mh] OR "Ultrasonography"[sh] OR Ultrasonics[mh] OR (echogra*[tiab] OR ultraso*[tiab] OR us guid*[tiab] OR us examin*[tiab] OR us imag*[tiab] OR preoperative-US[tiab] OR sonogra*[tiab] OR optoacoust*[tiab])) AND ("Diagnostic Errors"[mh] OR "diagnostic value"[mh] OR "sensitivity and specificity"[mh] OR "early diagnosis"[mh] OR "Reproducibility of Results"[mh] OR (diagnostic accurac*[tiab] OR diagnostic error*[tiab] OR diagnostic abilit*[tiab] OR diagnostic value*[tiab] OR false positive*[tiab] OR false negative*[tiab] OR predictive-value*[tiab] OR npv OR ppv OR sensitiv*[tiab] OR specific*[tiab] OR early diagnos*[tiab] OR early detect*[tiab] OR reproducib*[tiab] OR Misdiagnos*[tiab] OR observer varia*[tiab] OR interobserver varia*[tiab] OR intraobserver varia*[tiab] OR observer bias*[tiab] OR interobserver bias*[tiab] OR intraobserver bias*[tiab])) NOT (letter[pt] OR news[pt] OR comment[pt] OR editorial[pt] OR congresses[pt] OR abstracts[pt]) AND publisher[sb]

Google scholar n=100

melanoma "lymph|sentinel node|nodes" echography|ultrasound|ultrasonographic|ultrasonography "diagnostic|predictive accuracy|errors|ability|value"|misdiagnosis|"false positives|negatives"
